# A Case of Peripartum Cardiogenic Shock Resulting From Reverse Takotsubo Cardiomyopathy

**DOI:** 10.14740/jmc5324

**Published:** 2026-05-27

**Authors:** Nazish Tarar, Hannah Walsh, Katherine Sadaniantz, Shantel Brissett, Anna R. Whelan, Lara Kovell

**Affiliations:** a Department of Internal Medicine, UMass Chan Medical School, Worcester MA, USA; b UMass Chan Medical School, Worcester MA, USA; c Department of Cardiovascular Medicine, UMass Chan Medical School, Worcester MA, USA; d Department of Obstetrics and Gynecology, UMass Chan Medical School, Worcester MA, USA

**Keywords:** Takotsubo cardiomyopathy, Stress cardiomyopathy, Peripartum cardiogenic shock, Pregnancy

## Abstract

Takotsubo cardiomyopathy (TCM) is characterized by transient left ventricular (LV) dysfunction that classically presents as apical ballooning with basal hyperkinesis. A recognized variant, reverse TCM, conversely is characterized by basal and mid-ventricular hypokinesis with apical sparing, often triggered by a catecholamine surge. We present a case of a woman who underwent an emergency cesarean section complicated by the development of cardiogenic shock due to TCM. She had rapid clinical improvement with restoration of systolic function and was ultimately treated with guideline-directed medical therapy with a multidisciplinary team to ensure safety while lactating. In this case, the acute onset of LV systolic dysfunction in the setting of physical stress, wall motion abnormalities in a non-ischemic distribution, and rapid clinical recovery were factors that favored TCM as a diagnosis. The diagnosis of pregnancy-associated TCM is challenging, yet differentiation from other cardiomyopathies is important to guide acute management, as well as prognosis and counseling for subsequent pregnancies.

## Introduction

Takotsubo cardiomyopathy (TCM), also known as stress cardiomyopathy, is characterized by transient left ventricular (LV) dysfunction, in which wall motion abnormalities extend beyond a single epicardial artery distribution in the absence of significant coronary artery stenosis [1, 2]. While the pathophysiology of TCM is not completely understood, leading hypotheses suggest that a stress-induced catecholamine surge causes direct damage to cardiomyocytes, which in turn triggers LV dysfunction and coronary vasoconstriction, leading to ischemia without significant coronary artery obstruction [3]. The classic form of TCM presents with LV apical ballooning and basal hyperkinesis [3]. A recognized variant, known as reverse TCM, displays the opposite pattern, with basal and/or mid-ventricular hypokinesis and apical sparing [4]. While about 90% of patients with TCM are postmenopausal women [3], the physical and emotional stress surrounding childbirth creates the perfect milieu for the development of TCM [4].

TCM in the peripartum period is rare but increasingly recognized. A national study conducted in the United States reported a prevalence of approximately three cases per 100,000 pregnancy-associated hospitalizations. Notably, patients with TCM had a 98.7-fold increased risk of in-hospital mortality and were 14.7 times more likely to experience a prolonged hospital stay as compared with non-TCM pregnancy-associated hospitalizations [5]. These findings underscore the importance of timely diagnosis, appropriate management, and comprehensive counseling regarding the implications for future pregnancies.

This case highlights the diagnostic challenges associated with TCM, which is a diagnosis of exclusion and closely mimics other acute cardiac conditions such as peripartum cardiomyopathy (PPCM) and acute coronary syndrome (ACS). Furthermore, the difficulty in managing these patients safely during pregnancy or while lactating, where therapeutic options may be limited, further underscores the necessity of a multidisciplinary approach to management.

## Case Report

A 39-year-old G5P4 female at 39 weeks and 1 day of gestation, with beta-thalassemia trait, *in vitro* fertilization outside the USA, and limited prenatal care from 16 to 38 weeks of gestation, presented for a planned repeat cesarean delivery. She had no prior pregnancy-related complications. She had no acute complaints on presentation. Lab work completed 3 days prior to presentation showed a basic metabolic panel (BMP) and complete blood count (CBC) within normal limits, aside from mild anemia with a hemoglobin level of 10.8 g/dL (upper limit of normal 15.5 g/dL). Initial vitals were within normal limits, with blood pressure (BP) of 117/71 mm Hg and a heart rate (HR) of 99 beats per minute (bpm).

Following administration of combined spinal and epidural anesthesia for surgical analgesia, using lidocaine, bupivacaine, and fentanyl, the patient developed an acute, severe headache with BP 222/152 mm Hg and HR 124 bpm (sinus), concerning for hypertensive emergency, and so a one-time dose of intravenous labetalol 10 mg was administered. The patient then had a precipitous drop in BP, with a *nadir* of 77/63 mm Hg and a reduction in HR to 96 bpm, which resulted in fetal bradycardia. Thus, an emergency cesarean section was performed due to fetal instability. Estimated blood loss during the cesarean section was 830 mL.

**Figure 1:**
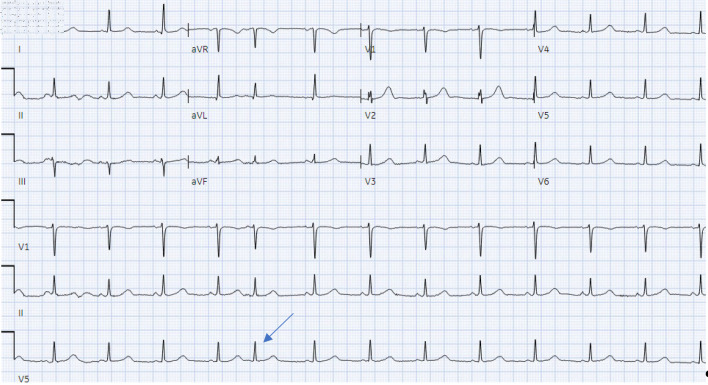
ECG revealing sinus rhythm and premature atrial complexes (arrow). ECG: electrocardiogram.

Postoperatively, the patient was persistently hypotensive, which was initially thought to be hypovolemic in etiology. The patient was fluid resuscitated with 6.1 L of lactated ringers and 250 mL of albumin with refractory hypotension. Given concern of a possible component of anesthesia-induced hypotension, vasopressor support was initiated with phenylephrine. The patient subsequently developed acute hypoxic respiratory failure, ultimately requiring 10 L supplemental oxygen. Given clinical decline, labs were drawn and demonstrated a lactic acid of 1.5 mmol/L (upper limit of normal 1.9 mmol/L), brain natriuretic peptide of 23 pg/mL (upper limit of normal 100 pg/mL), and troponin I trend of 0.29, 0.35, 0.51, and 0.33 ng/mL (upper limit of normal 0.04 ng/mL). Electrocardiogram (ECG) showed sinus rhythm with premature atrial complexes and no ischemic changes (Fig. 1). Stat portable chest X-ray revealed an enlarged cardiac silhouette with pulmonary vascular congestion. Intravenous diuresis was initiated, and due to concern for cardiogenic shock, the cardiology service was emergently consulted while the patient was in the post-anesthesia care unit. Bedside transthoracic echocardiography (TTE) showed a dilated cardiomyopathy with a left ventricular ejection fraction (LVEF) of 30%, LV diastolic volume of 160.2 mL, LV systolic volume 110.1 mL, diffuse hypokinesis of the basal-mid walls with apical sparing, and moderate mitral regurgitation (MR). At this point, diagnostic considerations included reverse TCM and PPCM. The acute onset of LV dysfunction immediately following emergency cesarean delivery, a well-defined physiologic stressor, the TTE pattern of diffuse hypokinesis with apical sparing in a non-ischemic distribution, and the absence of cardiac symptoms during the preceding weeks of pregnancy favored TCM. PPCM would have likely demonstrated a more gradual onset of symptoms over the course of a month with global LV hypokinesis on TTE.

The patient was transferred to the cardiac care unit (CCU) for intravenous diuresis and inotropic support with dobutamine (initiated at 2.5 µg/kg/min), given the concern for cardiogenic shock. Phenylephrine was transitioned to norepinephrine at 0.04 µg/kg/min, but this was quickly weaned within 2 h of dobutamine initiation as hypotension rapidly improved. Dobutamine was weaned and discontinued within 48 h. Guideline-directed medical therapy (GDMT) was initiated. Spironolactone 25 mg daily was started while the patient was on a reduced dose of dobutamine; metoprolol succinate 25 mg daily was initiated the day after dobutamine was discontinued; and enalapril 5 mg daily was initiated 3 days later. Repeat TTE was performed 3 days after delivery and demonstrated normalized LV size, improved LVEF of 51%, and trace MR. Spironolactone was discontinued given a low normal EF. The transient regional wall motion abnormalities and rapid recovery of LV function (LVEF 30% to 51% in 3 days) favored TCM as the diagnosis. The patient was ultimately discharged 7 days after delivery on enalapril and metoprolol succinate (Table 1). The patient continued to breastfeed while on her prescribed medications. Four months following delivery, repeat TTE revealed an LVEF 55–60%. She was continued on monotherapy with metoprolol succinate.

**Table 1:** Clinical Timeline

Timepoint	Phase	Events and interventions	Key findings
Initial presentation	Perioperative	Planned repeat cesarean section under combined spinal and epidural anesthesia	No complaints; vitals unremarkable
Combined spinal-epidural anesthesia administration	Perioperative	Hypertensive emergency + severe headache → IV labetalol 10 mg → hypotension → fetal bradycardia → emergency cesarean (EBL 830 mL)	Maternal BP 222/152 mm Hg→ hypotension nadir 67/36 mm Hg
PACU	Acute decompensation	Persistent hypotension refractory to 6.1 L LR + 250 mL albumin → phenylephrine initiated Acute hypoxic respiratory failure requiring 10 L O_2_; maternal fetal medicine consulted; diuresis started	Lactate 1.5 mmol/L; BNP 23 pg/mL; troponin I 0.29 → 0.35 → 0.51 ng/mL; ECG: sinus rhythm with PACs, no ischemic changes; CXR: enlarged cardiac silhouette and signs of pulmonary vascular congestion
PACU	Acute decompensation	Emergent cardiology consult → bedside TTE performed	LVEF 30%; diffuse hypokinesis with apical sparing; moderate MR
CCU admission	Acute decompensation	Transferred to CCU; dobutamine 2.5 µg/kg/min initiated → hemodynamic improvement; phenylephrine transitioned to norepinephrine	Improved hemodynamics on inotropic support
CCU day 1–2	Stabilization and GDMT	Spironolactone 25 mg daily initiated; dobutamine weaned and discontinued within 48 h	Troponin I 0.33 ng/mL; continued hemodynamic improvement
CCU day 3	Stabilization and GDMT	Metoprolol succinate 25 mg daily started (day after dobutamine discontinued); repeat TTE performed → spironolactone discontinued	Troponin I 0.09 ng/mL; normal LV size; LVEF 51%; trace MR
CCU day 6	Stabilization and GDMT	Enalapril 5 mg daily initiated	Continued clinical improvement; breastfeeding maintained on GDMT
Day 7–discharge	Recovery	Discharged home on enalapril + metoprolol succinate; patient continued breastfeeding	LVEF 51% at discharge
4-month follow-up	Recovery	Continued on metoprolol succinate monotherapy (enalapril discontinued given normalized EF)	LVEF 55–60%

BP: blood pressure; BNP: brain natriuretic peptide; CCU: cardiac care unit; CXR: chest X-ray; EBL: estimated blood loss; ECG: electrocardiogram; GDMT: guideline-directed medical therapy; LR: lactated Ringer’s; LV: left ventricle; LVEF: left ventricular ejection fraction; MR: mitral regurgitation; O_2_: oxygen; PACs: premature atrial complexes; PACU: post-anesthesia care unit; TCM: takotsubo cardiomyopathy; TTE: transthoracic echocardiography.

## Discussion

Given the associated increased risk of prolonged hospital stay, in-hospital mortality, and overlapping features with other clinical syndromes, there is considerable importance placed on the identification and management of TCM [6]. Particularly in the peripartum period, TCM presents a diagnostic and management challenge, requiring a multidisciplinary approach to ensure the safety of both mother and child.

Differentiating TCM from other cardiomyopathies, particularly PPCM, demands careful consideration in order to understand prognosis and provide further counseling. A 2022 review elucidated distinguishing features of TCM in the postpartum period compared to PPCM (Table 2) [7–10]. TCM often presents more rapidly and over a shorter timeframe immediately after delivery in the setting of a clear emotional or physiological trigger, whereas PPCM develops more insidiously within the last few months of pregnancy and up to 6 months following delivery without a discrete precipitant.

**Table 2:** Distinguishing Characteristics of Takotsubo CM and Peripartum CM

	Takotsubo CM	Peripartum CM
Echocardiographic findings	Transient regional wall motion abnormalities, most often apical ballooning	Global left ventricular hypokinesis
Timeline	Occurs within the days surrounding delivery	Can occur within the months surrounding delivery
Left ventricular function prognosis	More favorable prognosis of LVEF recovery	Less favorable prognosis of LVEF recovery
Recovery	Quicker recovery of LVEF, within 1 month (75% of patients recover within 10 days) [8]	Delayed recovery of LVEF
Incidence	Higher incidence (1 in 5,000 admissions)	Lower incidence (1 in 1,000–4,000 live births)
Management	Supportive management with GDMT	Heart failure management with GDMT

Adapted from Tzerefos et al [9] and Garg et al [10]. CM: cardiomyopathy; LVEF: left ventricular ejection fraction; GDMT: guideline-directed medical therapy.

In this case, the immediate temporal relationship with the emergency cesarean delivery and the onset of LV dysfunction strongly favored TCM. Data from the prospective nationwide RETAKO registry revealed that patients with peripartum-associated TCM had a higher prevalence of dyspnea, with atypical symptoms and ECG patterns including ST-segment elevation compared to TCM not associated with the peripartum period [8]. On TTE, TCM is most often characterized by regional wall motion abnormalities in a non-coronary distribution, typically with apical ballooning during systole and diastole, while PPCM displays global LV hypokinesis during systole and diastole [4, 7]. The pattern of hypokinesis of the basal-mid walls with apical sparing seen in this patient is consistent with the reverse TCM variant, which typically involves basal-mid wall hypokinesis to akinesis. This pattern did not fit the global dysfunction typical of PPCM [4]. Additionally, patients with TCM typically show a full recovery of LVEF within 1 month, whereas PPCM has a much more delayed LV function recovery [2, 4, 7]. Some studies have suggested that only 23–41% of patients with PPCM develop complete recovery of LVEF, whereas patients with TCM most often recover within days to weeks [7, 9, 10]. In our patient, normalization of LVEF from 30% to 51% within 3 days and 55–60% at 4 months is consistent with the expected recovery course of TCM and would be atypical for PPCM. Given the longer course for LV function recovery in PPCM, it carries higher rates of cardiovascular complications than TCM, including complex ventricular arrhythmias, chronic congestive heart failure, and mortality of 10–28% at 6 months and 2 years, respectively [7]. Accurate differentiation therefore has direct implications not only for prognosis but also for counseling regarding subsequent pregnancies.

ACS was also on the differential in this case, but, given the lack of ischemic changes in a territorial pattern on ECG and a modest troponin trend relative to the degree of dysfunction, this was felt to be less likely. Furthermore, wall motion patterns on echocardiography did not correspond to a single epicardial vessel distribution, as would typically be suggestive. Rapid recovery of LVEF also favored TCM. Thus, further evaluation of coronary anatomy via CT angiography or invasive coronary angiography was deferred.

Both TCM and PPCM pose difficulties in management, as most components of GDMT pose teratogenic risks. Angiotensin-converting enzyme (ACE) inhibitors and angiotensin II receptor blockers (ARBs), essential pharmacological treatments demonstrated to effectively reduce the recurrence of stress cardiomyopathy, are contraindicated in pregnancy due to an increased risk of major congenital cardiovascular anomalies, stillbirth, oligohydramnios, pulmonary hypoplasia, limb defects, fetal growth restriction, miscarriage, and preterm birth [4]. Other pillars of GDMT include mineralocorticoid receptor antagonists and sodium-glucose cotransporter 2 (SGLT2) inhibitors, which are also contraindicated during pregnancy due to concerns about feminization of male fetuses and renal dysfunction, respectively [11, 12]. Thus, peripartum treatment is focused on diuretics, beta blockers, afterload reduction with hydralazine or nitrates, and supportive care [11].

In the postpartum period, the addition of ACE inhibitors, ARBs, and mineralocorticoid receptor antagonists allows for more complete GDMT. For patients who are breastfeeding, medication selection should be guided by established lactation safety data. Enalapril is the preferred ACE inhibitor due to negligible breast milk levels, undetectable infant plasma levels, and absence of reported adverse effects in breastfed infants [12]. Metoprolol succinate is among the beta-blockers of choice during lactation, as only small amounts are excreted into breast milk, and no adverse reactions have been documented in breastfed infants [12]. Studies have shown that breastfeeding infants receive approximately 0.2% of maternal daily exposure to canrenone, the active metabolite of spironolactone, making it compatible with breastfeeding [12]. Furosemide and other diuretics are also generally safe for use during lactation [12]. For patients planning to breastfeed, counseling regarding the safety of medications should be conducted using evidence-based tools such as LactMed or in consultation with a maternal fetal medicine physician or obstetrician gynecologist. Cessation of lactation or “pump and dump” is not recommended for the large majority of patients.

Several studies have investigated the recurrence rate for TCM, with estimates varying widely, between 1% and 11.4% [13, 14]. In a study conducted by Santoro et al, there was no significant clinical evidence suggesting that the chronic management of TCM reduced the risk of recurrence [13]. Other studies have suggested that it is the trigger that affects prognosis, and that emotionally triggered TCM has more favorable short- and long-term prognosis compared to TCM secondary to neurologic disorders, physical activities, medical conditions, or procedures [15]. We hypothesize that, in our case, pain, anxiety, stress, and perhaps side effects of analgesia (as bupivacaine can cause hypertension in rare cases) led to hypertensive emergency in our patient [16, 17]. Due to impaired baroreceptor reflex function, which is known to be depressed during pregnancy, administration of labetalol and maternal hypotension led to a rapid swing in BP, leading to significant hypotension with impact on fetal health [16, 17]. Given the significant physiologic stress, including rapid fluctuations in BP and blood loss anemia, as well as emotional stress induced by this pregnancy, a catecholamine surge is suspected to have had direct myocardial stunning effects on beta adrenergic receptors [18]. Furthermore, labetalol blocks α1-, β1-, and β2-adrenoreceptors. The ratio of α- to β-blockade has been estimated to be approximately 1:7, which is greater when compared to other non-selective beta blockers such as carvedilol. This greater alpha-blocking effect may cause more hypotension, a significant concern in pregnant patients who may already be hemodynamically compromised [18].

Outcomes in subsequent pregnancies in patients with a history of peripartum TCM are not well known, likely due to the rarity of the condition. Nonetheless, thorough preconception counseling should be completed and include an evaluation of cardiac function with echocardiography. Subsequent monitoring throughout pregnancy should include serial echocardiography, N-terminal pro-brain natriuretic peptide monitoring, and continuation of beta-blocker therapy [19]. Timely initiation of contraception in postpartum individuals with TCM is critical in order to allow for recovery and prevention of short-interval pregnancy. Methods including long-acting reversible contraception (levonorgestrel or copper-containing intrauterine devices or the etonogestrel implant), progestin only oral contraceptives, and barrier methods are all safe for individuals who have cardiomyopathy. Per the United States Medical Eligibility Criteria (MEC) guidelines, combined oral contraceptives should be avoided in patients with cardiomyopathy, irrespective of time elapsed since the index event and the degree of cardiac dysfunction [20].

Although there are no specific guidelines for subsequent pregnancies after peripartum TCM, there are risk stratification guidelines based on LV function recovery that can be used to counsel these patients. The 2022 American Heart Association (AHA)/American College of Cardiology (ACC)/Heart Failure Society of America (HFSA) Heart Failure Guidelines highlight pre-pregnancy LVEF as the strongest predictor of outcomes in subsequent pregnancies [21]. Women with persistent LV dysfunction with LVEF less than 50% should be strongly discouraged from pregnancy due to increased risk of worsening LVEF and worse fetal outcomes [21]. Additional risk factor stratification and modification should be completed, including evaluation of other comorbidities such as hypertension, underlying anxiety or psychological stress, and diabetes. Discussions between the clinician and the patient should be transparent regarding the uncertain risk of recurrence and the potential for adverse fetal and maternal outcomes. Multidisciplinary management with maternal-fetal medicine, cardiology, and anesthesiology is essential should a patient have a subsequent pregnancy, in addition to close surveillance and echocardiographic monitoring.

## Data Availability

The authors declare that data supporting the findings of this study are available within the article.
